# Supplementary material for: Cell Differentiation and Proliferation in the Bone Marrow and Other Organs of 2D2 Mice during Spontaneous Development of EAE Leading to the Production of Abzymes
Source: Molecules. 2022 Mar 28;27(7):2195. doi: 10.3390/molecules27072195 (PMC9000721; doi:10.3390/molecules27072195)
Supplement: Supplementary file 1 [file molecules-27-02195-s001.zip › molecules-1647268-supplementary.pdf]

Supplementary Materials

Cell Differentiation and Proliferation in the Bone Marrow and Other Organs of 2D2 Mice during Spontaneous Development of EAE Leading to the Production of Abzymes

Kseniya S. Aulova [1], Andrey E. Urusov [1], Ludmila B. Toporkova [2], Sergey E. Sedykh [1], Juliya A. Shevchenko [2], Valeriy P. Tereshchenko [2], Sergei V. Sennikov [2], Irina A. Orlovskaya [2] and Georgy A. Nevinsky [1,*]

1. Institute of Chemical Biology and Fundamental Medicine, Siberian Branch of the Russian Academy of Sciences, 630090 Novosibirsk, Russia
2. Institute of Clinical Immunology, Siberian Branch of the Russian Academy of Sciences, 630090 Novosibirsk, Russia

*****Correspondence: nevinsky@niboch.nsc.ru

Compliance with stringent criteria was verified according to [13,14,34-42].

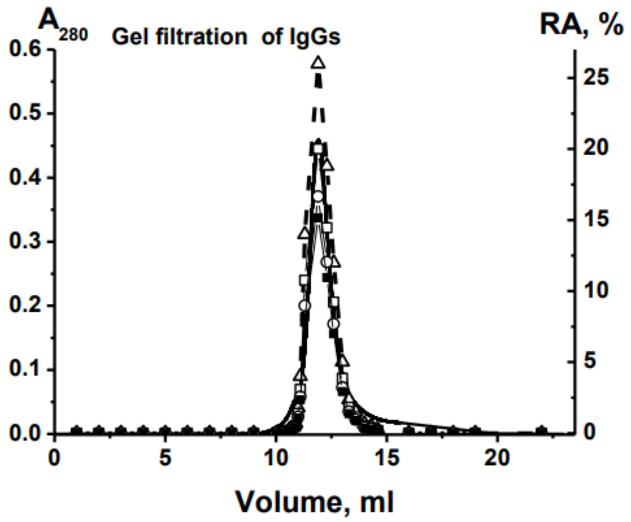

Supplementary Figure S1.

Application of the strict criteria to prove that the DNA-, MBP-, MOG- and histone-hydrolyzing activities IgG_{mix} are intrinsic property of IgG_{mix}. FPLC gel filtration IgG_{mix} on a Superdex 200 column in an acidic buffer (pH 2.6) after Abs incubation in the same buffer: (—), absorbance at 280 nm (A_{280}); relative activity (RA) of IgG_{mix} in the hydrolysis of DNA (Δ), MOG (□), MBP (•), and histones (o). A complete hydrolysis of substrates for 10 h was taken for 100%. The error in the initial rate determination from two experiments in each case did not exceed 7–10%.

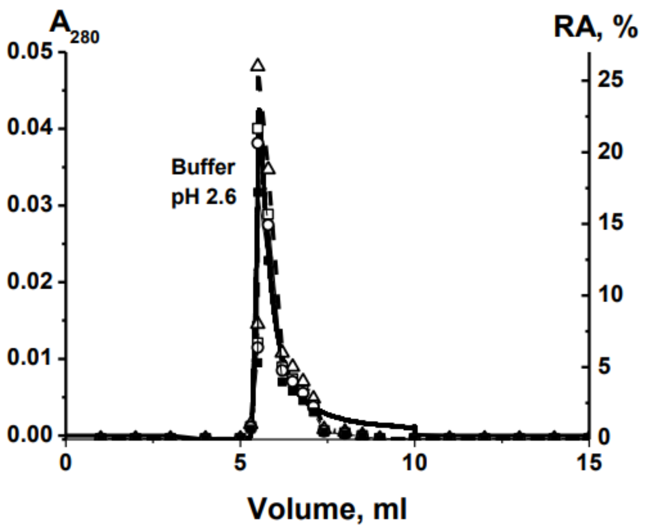

Supplementary Figure S2.

Application of the strict criteria to prove that the DNA-, MBP-, MOG- and histone-hydrolyzing activities IgG_{mix} are intrinsic property of IgG_{mix}. Affinity chromatography of the IgG_{mix} on Sepharose bearing rabbit IgGs against of mouse IgGs: (—), absorbance at 280 nm (A_{280}); relative activity (RA) of IgG_{mix} in the hydrolysis of DNA (Δ), MOG (□), MBP (•), and histones (o). A complete hydrolysis of substrates for 10 h was taken for 100%. The error in the initial rate determination from two experiments in each case did not exceed 7–10%.

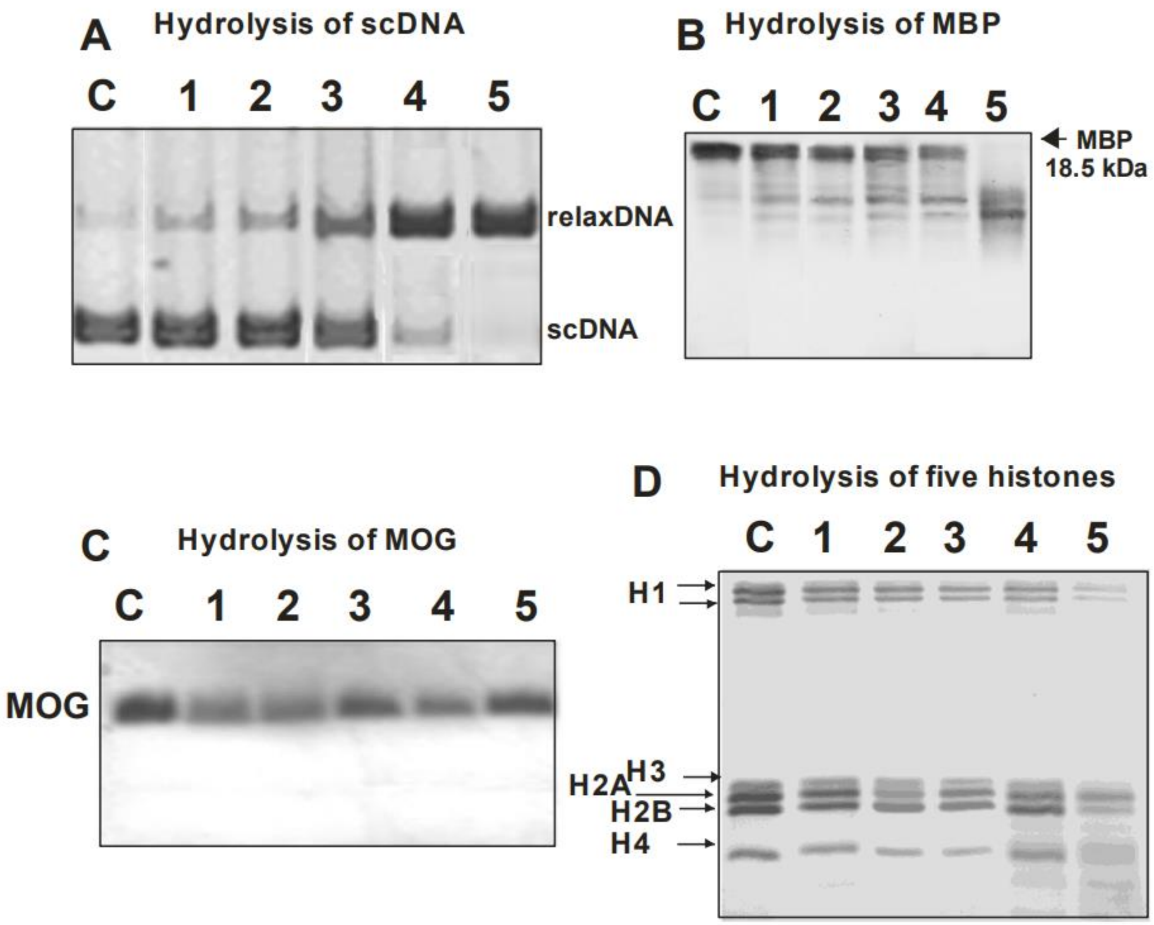

Supplementary Figure S3.

The DNase and protease activities of antibodies were analyzed according to [13,14,34-42]. As examples, the Figure shows typical data of analysis of the relative DNase activity by agarose electrophoresis (**A**), estimation of MBP- (**B**), MOG- (**C**), and histones-hydrolyzing (**D**) activities of IgGs from the blood of five individual 2D2 mice using SDS-PAGE. Lanes C correspond to these substrates incubated in the absence of IgGs. Supercoiled (sc) DNA (20 μg/ml) was incubated for 4 h (0.05 mg/ml IgGs), while MBP (1.0 mg/ml), MOG (0.5 mg/ml) and mixture of 5 histones (1.0 mg/ml) were incubated in the absence or in the presence of 0.05 mg/ml IgGs for 24 h. The error in the initial rate determination from 2-3 experiments in the case of every mouse of each group did not exceed 7–10%. For other details see Methods.

Affinity chromatography of the sle-IgG_{mix} on Sepharose bearing mouse IgGs against light chains of human IgGs (A) and FPLC gel filtration sle-IgG_{mix} on a Superdex 200 column in an acidic buffer (pH 2.6) after Abs incubation in the same buffer (B): (—), absorbance at 280 nm (A_{280}); (□), relative activity (RA) of sle-IgG_{mix} in the hydrolysis of hMBP. A complete hydrolysis of 0.5 mg/ml hMBP for 24 h was taken for 100%. The error in the initial rate determination from two experiments in each case did not exceed 7–10%.
